# Evaluation of a Rapid and Simplified Protocol for Direct Identification of Microorganisms From Positive Blood Cultures by Using Matrix Assisted Laser Desorption Ionization Time-of-Flight Mass Spectrometry (MALDI-TOF MS)

**DOI:** 10.3389/fcimb.2021.632679

**Published:** 2021-03-11

**Authors:** Yufeng Dai, Xinyi Xu, Xue Yan, Daming Li, Wei Cao, Lingli Tang, Min Hu, Chuanhao Jiang

**Affiliations:** ^1^Department of Laboratory Medicine, The Second Xiangya Hospital, Central South University, Changsha, China; ^2^Center for Experimental Medicine, The Third Xiangya Hospital, Central South University, Changsha, China; ^3^Clinical Molecular Diagnostic Center of Hunan Province, The Second Xiangya Hospital, Central South University, Changsha, China

**Keywords:** bloodstream infections, blood culture, MALDI‐TOF MS, direct identification, optimized protocol

## Abstract

Early and rapid identification of microorganisms is critical for reducing the mortality rate caused by bloodstream infections (BSIs). The accuracy and feasibility of directly identifying pathogens in positive blood cultures by matrix assisted laser desorption ionization time-of-flight mass spectrometry (MALDI-TOF MS) has been intensely confirmed. In this study, we combined density centrifugation and extra chemical lysis-extraction to develop an optimized method in the blood culture process, which significantly improved the effectiveness of direct identification by MALDI-TOF MS. The accuracy was evaluated by 2,032 positive blood culture samples (115 species of microorganism). The overall MALDI-TOF MS based identification rate with scores ≥ 1.700 was 87.60%. 94.06% of gram-negative bacteria were identified consistently to the genus level, followed by anaerobes (93.33%), gram-positive bacteria (84.46%), and fungi (60.87%). This protocol could obtain results within 10–20 min at a cost of less than $0.1 per sample, which saved up to 24 h in identifying 87.60% of the microorganism from positive blood cultures. This rapid and simplified protocol facilitates the direct identification of microorganism in positive blood cultures, and exhibits the advantages of cost-effective, time-saving, and easy-to-use. It could provide the causative organism of the patient to clinicians in time for targeted treatment and reduce mortality.

## Introduction

Bloodstream infections (BSIs) are the major cause of sepsis-related morbidity and mortality in hospitalized patients worldwide (Kumar et al., 2006; Tabah et al., 2012; Mortality and Causes of Death, 2015). The 58.3% of the nosocomial BSIs in the intensive care unit (ICU) were caused by gram-negative bacteria, 32.8% by gram-positive bacteria, 7.8% by fungi, and 1.2% by obligate anaerobes (Tabah et al., 2012). The mortality rate of sepsis-related hypotensive patients is increasing at a rate of 7.6% per hour (Kumar et al., 2006). Hence, rapid identification of the causative organism is crucial for the clinical treatment of BSIs and decreasing mortality.

Blood culture remains the reference standard for the diagnosis of BSIs (Peker et al., 2018). The traditional identification process requires culture broth from the positive blood culture bottles to be streaked on solid media and incubated for 18–24 h. The pure colonies were obtained from those subculture media for subsequent pathogen identification and antimicrobial sensitivity testing (AST). Although this traditional method is useful for routine identification of general organisms, it is challenging for fastidious bacteria such as anaerobes (Dubourg and Raoult, 2016). Moreover, the long turn-around time (TAT) would greatly depreciate its value as a rapid diagnostic method.

Matrix-assisted laser desorption ionization–time-of-flight mass spectrometry (MALDI-TOF MS) combines the MALDI source and the TOF mass. It can identify bacteria based on comparing the protein profiles of bacteria with standard profiles of known bacteria in a database (Angeletti, 2017). MALDI-TOF MS has been proved to be a high-throughput and efficient microbial identification system, which improves the reliability of microbial identification and reduces the complexity of operation (Martin, 2012; Gorton et al., 2014; Doern and Butler-Wu, 2016; Hou et al., 2019). It has been widely utilized in clinical microbiology laboratories, especially for conventional identification of pure colony cultured on solid mediums and direct identification of microorganism in blood cultures. Although notable efforts have been made, the existing methods are still relatively time-consuming and laborious, which limits the clinical application of MALDI-TOF MS in rapid diagnosis (Gorton et al., 2014; Jakovljev and Bergh, 2015; Robinson and Ussher, 2016). Also, nonbacterial proteins in blood culture broth disturb the analysis of microbial proteome profiles during direct identification. Therefore, a rapid and effective preprocessing method to eliminate interference proteins while also concentrating the bacterial is still explored. It could facilitate the widespread application of MALDI-TOF MS for the direct identification of microorganisms from blood cultures (Di Gaudio et al., 2018; Scohy et al., 2018).

**Figure 1:**
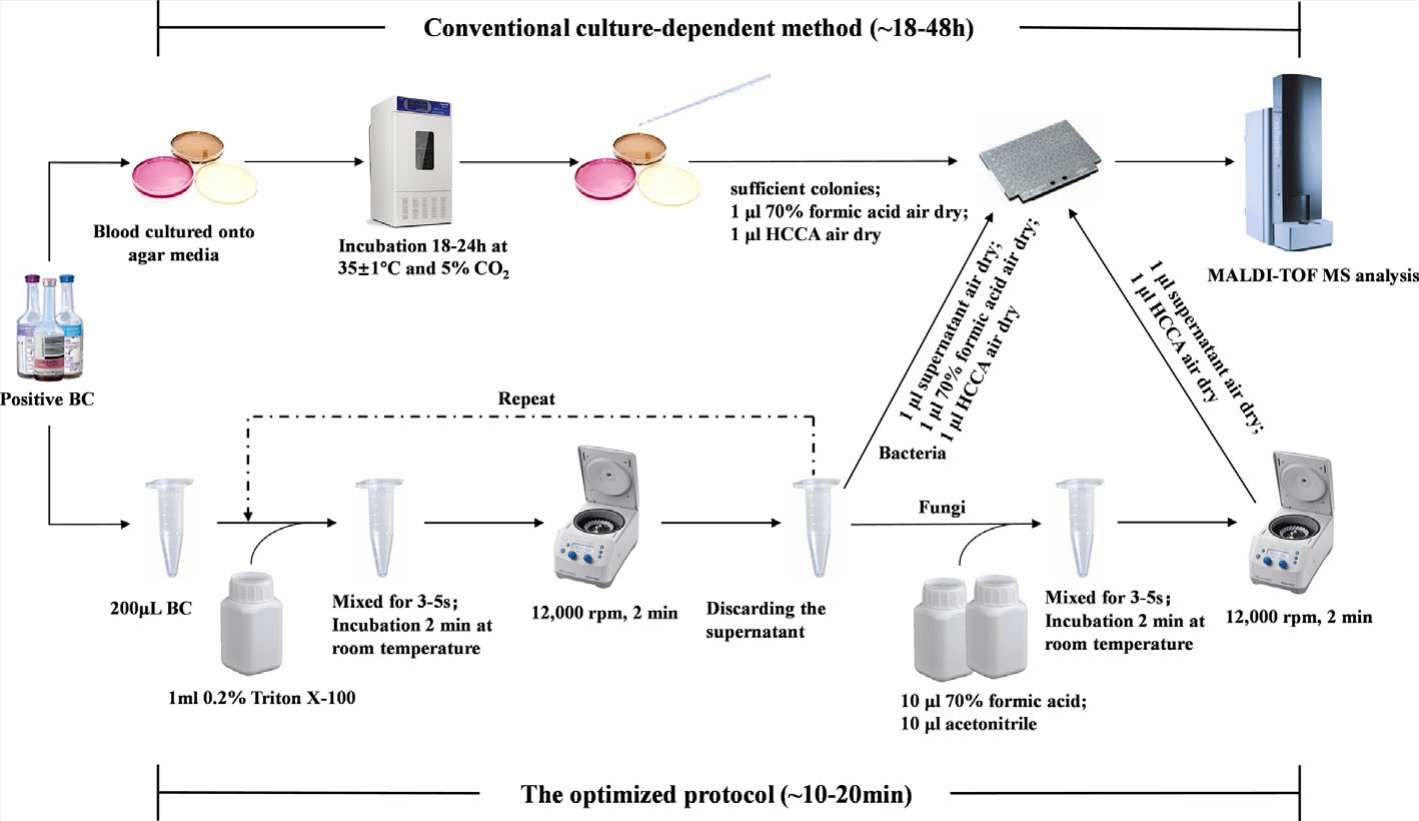
Workflow of direct identification in positive blood cultures using MALDI-TOF MS.

This study aims to develop a relatively cost-effective and simplified protocol to directly identify microorganisms from positive blood cultures with high reliability. We optimized the previous pretreatment protocol, and prospectively assessed the performance of this protocol by comparing it with the conventional culture-dependent identification method. The complete workflow was shown in **Figure 1**. Our protocol would contribute to promptly provide causative organism of patients to clinicians for appropriate antimicrobial therapy promptly and reduce mortality.

## Materials and Methods

### Sample Collection

2,081 blood culture bottles were detected positive from the Second Xiangya Hospital of Central South University between October 2018 to October 2019. 32 of the positive cultures failed to grow on subculturing, and 17 were polymicrobial. Those samples were excluded from the study. For patients with multiple blood cultures simultaneously, only the blood culture that was first reported to be positive was used for further experiments to avoid repetition. Finally, the remaining 2,032 monomicrobial blood cultures, belonging to 1,870 adult patients and 162 pediatric patients, were included in the study. This study has been approved by the research and ethics committee of the Second Xiangya Hospital of Central South University. All patients are anonymized and no results were used in patient management. Therefore, no informed consent was required.

### Blood Culture Processing

Blood was taken with aseptic technique, directly inoculated into aerobic (BD BACTEC Plus Aerobic/F) and anaerobic culture vials (BD BACTEC Lytic/10 Anaerobic/F), or peds culture vials (BD BACTEC Peds Plus/F culture vials) (Becton Dickinson & Co, BD, Shanghai, China). Each adult patient needed an Aerobic and an anaerobic culture vials, the Peds vials were used only for pediatric patients. All blood culture bottles were loaded onto the BD BACTEC FX instrument (Becton Dickinson, Franklin Lakes, NJ, USA).

### Conventional Culture-Dependent Identification Method

Upon signaling positive, the blood culture broth was analyzed by Gram stain and cultured onto a blood agar plate (BIOIVT, Zhengzhou, China) and/or obligate anaerobic agar plate (BIOIVT, Zhengzhou, China), and incubated in 5%CO_2_ at 35 ± 1°C for 18 to 24 h (Thermo Fisher Scientific, USA). Whenever Gram stain indicated the presence of fungi, the sample was additionally subcultured on SDA agar plate (BIOIVT, Zhengzhou, China) and incubated at 37°C for 48 h. Following incubation, a sterile, disposable inoculation loop was used to transfer sufficient colonies of a pure culture from those subculture media to a 96-spot polished steel target plate (Bruker Daltonics, Bremen, Germany) for MALDI-TOF MS analysis.

### Lysis and Centrifugation

The blood culture bottle was vigorously shaken to ensure homogeneous mixing. 200 μl blood culture broth were harvested from positive blood culture and added into a 1.5 ml Eppendorf tube. 1 ml solution of Triton X-100 (Solarbio Biotech, Beijing, China) at a concentration of 0.2% were added. The mixture was vortexed briefly and then incubated at room temperature for 2 min. Following centrifuged at 12,000 rpm for 2 min, the supernatant was discarded, and a further 1 ml of 0.2% Triton X-100 was added before a second cycle of vortexing and centrifugation. The supernatant was discarded again, and the upper liquid was carefully removed to retain the white precipitation. Then, one loop of the white precipitation was picked out by a sterile, disposable inoculation loop and deposited onto a 96-spot polished steel target plate for MALDI-TOF MS analysis.

### Extra Extraction for Fungi

The precipitation obtained in the previous step was re-suspended in 10 μl of 70% formic acid (Sigma-Aldrich, Shanghai, China). The mix was vortexed for 5 s and then incubated at room temperature for 2 min. Ten microliters of acetonitrile (Sigma-Aldrich) was added before a second cycle of vortexing and centrifugation. Finally, 1 μl of the supernatant was deposited onto a 96-spot polished steel target plate for MALDI-TOF MS analysis.

### MALDI-TOF MS

After drying the bacterial pellet on a MALDI‐TOF MS target plate at room temperature, 1 μl of 70% formic acid (70% v/v) was added to each spot and air-dried (fungal pellets after an extra extraction could skip this step). Lastly, 1 μl of alpha‐cyano‐4‐hydroxycinnamic acid (HCCA) matrix solution was placed onto each spot and then air‐dried for MALDI-TOF analysis.

Bruker LT Microflex MALDI-TOF MS (Bruker, Daltonics, Germany), Bruker Biotyper 2.3 system software, and Bruker database 5989 were adopted to read the target plates. The mass spectrometer was calibrated using a Bruker BTS (bacterial test standard) spot: *Escherichia coli* and three internal control spots: *Escherichia coli* ATCC 25922, *Staphylococcus aureus* ATCC 25923, and *Candida albicans* ATCC 90028. After analysis with Microflex LT, Biotyper software calculated a similarity score [log(score)] by comparing the protein spectra of each spot with the database spectra. Ten scores per spot could be obtained, ranging from a higher to lower probability of valid identification. According to the manufacturer’s instructions, a score ≥2.000 indicates identification to the species level, a score between 1.700 and 1.999 indicates identification to the genus level, and a score <1.700 indicates no reliable identification. The inconsistent identification between the conventional culture-dependent method and optimized protocol was further characterized by 16S rRNA gene sequencing at the reference laboratory.

## Results

**Figure 2:**
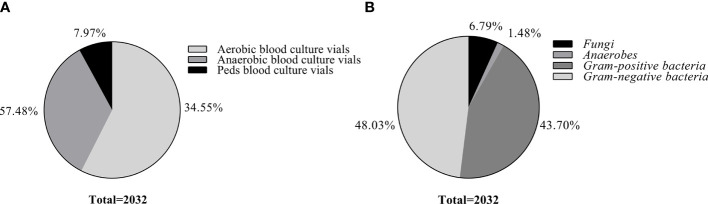
Distribution of blood culture vials and different microbial groups identified by conventional culture-dependent method. **(A)**. Distribution of blood culture vials used in this study. **(B)**. Distribution of different microbial groups identified by conventional culture-dependent method.

**Figure 3:**
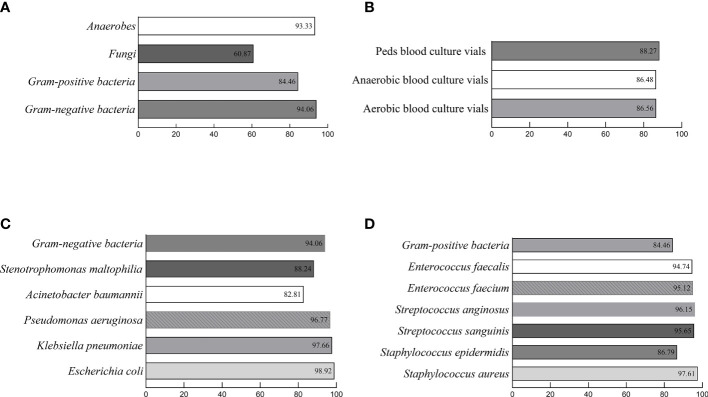
Consistency rates of the optimized protocol for direct identification of microorganisms from positive blood cultures with the conventional method. **(A)**. The percentages of concordant results were 93.33%/60.87%/84.46%/94.06%, respectively for Fungi, Anaerobes, Gram-positive bacteria, and Gram-negative bacteria with a log(score) of ≥1.700. **(B)**. The percentages of concordant results were 88.27%/86.48%/86.56%, respectively for Peds/Anaerobic/Aerobic blood culture vials with a log(score) of ≥1.700. **(C)**. The percentages of concordant results were 88.24%/82.81%/96.77%/97.66%/98.92%, respectively for general gram-negative bacteria *Stenotrophomonas maltophilia*, *Acinetobacter baumannii*, *Pseudomonas aeruginosa*, *Klebsiella pneumonia*, and *Escherichia coli* with a log(score) of ≥1.700. **(D)**. The percentages of concordant results were 94.74%/95.12%/96.15%/95.65%/86.79%/97.61%, respectively for general gram- positive bacteria *Enterococcus faecalis*, *Enterococcus faecium*, *Streptococcus anginosus*, *Streptococcus sanguini*, *Staphylococcus epidermidis*, and *Staphylococcus aureus* with a log(score) of ≥1.700.

In this study, a total of 2,032 positive blood cultures [57.48% (1,168/2,032) aerobic blood culture vials, 34.55% (702/2,032) anaerobic blood culture vials, and 7.97% (162/2,032) Peds blood culture vials] were collected after excluding 32 bacteria‐free blood cultures and 17 polymicrobial blood cultures (**Figure 2A**). All samples were classified based on the identification of the conventional culture-dependent method. 48.03% (976/2,032) strains were gram-negative bacteria, 43.7% (888/2,032) strains were gram-positive bacteria, 6.79% (138/2,032) strains were fungi, and 1.48% (30/2,032) strains were anaerobes (**Figure 2B**). The 115 different microbial species were isolated, and the effectiveness of the optimized method was evaluated by comparing with the conventional culture-dependent method under different log(score) threshold (**Table 1**). Without considering the cut-off value, the total coincidence rate of direct identification was 97.39% (1,979/2,032) compared to the conventional method. When setting the cut-off threshold to 1.700, the coincidence rate was 87.60% (1,780/2,032). More precisely, we identified 94.06% (918/976) gram-negative bacteria, 93.33% (28/30) anaerobes, 84.46% (750/888) gram-positive bacteria, and 60.87% (84/138) fungi to the genus level (**Figure 3A** and **Table 2**). The most common isolates from BSIs in this study were *Escherichia coli* (18.21%, 370/2,032), *Klebsiella pneumoniae* (10.53%, 214/2,032), and *Staphylococcus aureus* (10.29%, 209/2,032), which had high concordance rates of 98.92% (366/370), 97.66% (209/214), and 97.61% (204/209) respectively. The identification rates of different culture bottles were similar. The Peds blood culture bottles (88.27%, 143/162) had an excellent identification rate, followed by aerobic blood culture bottles (86.56%, 1,011/1,168), and anaerobic blood culture bottles (86.48%, 607/702) (**Figure 3B**).

**Table 1:** Consistency rate of the conventional culture-dependent method and the optimized method for identifying positive blood cultures by MALDI‐TOF MS (n = 2032).

Microorganisms	Conventional method (no. of isolates)	Direct method				Mis-identification (%)
		No. (%) log(score) of:				
		Score ≥2.000	Score ≥1.700	Score ≥1.400	Score <1.400	
***Gram-positive bacteria***	888	436 (49.10)	750 (84.46)	849 (95.61)	8 (0.90)	31 (3.49)
***Staphylococcus***	586	346 (59.04)	521 (88.91)	564 (96.25)	7 (1.19)	15 (2.56)
*Staphylococcus aureus*	209	174 (83.25)	204 (97.61)	206 (98.56)	1 (0.48)	2 (0.96)
*Staphylococcus auricularis*	1		1 (100.00)	1 (100.00)		
*Staphylococcus capitis*	33	17 (51.52)	29 (87.88)	29 (87.88)	3 (9.09)	1 (3.03)
*Staphylococcus caprae*	1	1 (100.00)	1 (100.00)	1 (100.00)		
*Staphylococcus cohnii*	2					2 (100.00)
*Staphylococcus epidermidis*	159	59 (37.11)	138 (86.79)	153 (96.23)	1 (0.63)	5 (3.14)
*Staphylococcus equorum*	1	1 (100.00)	1 (100.00)	1 (100.00)		
*Staphylococcus haemolyticus*	43	9 (20.93)	29 (67.44)	41 (95.35)	1 (2.33)	1 (2.33)
*Staphylococcus hominis*	117	76 (64.96)	108 (92.31)	116 (99.15)		1 (0.85)
*Staphylococcus intermedius*	2					2 (100.00)
*Staphylococcus lentus*	1					1 (100.00)
*Staphylococcus lugdunensis*	10	7 (70.00)	7 (70.00)	9 (90.00)	1 (10.00)	
*Staphylococcus pettenkoferi*	1	1 (100.00)	1 (100.00)	1 (100.00)		
*Staphylococcus saprophyticus*	3			3 (100.00)		
*Staphylococcus sciuri*	1			1 (100.00)		
*Staphylococcus warneri*	2	1 (50.00)	2 (100.00)	2 (100.00)		
***Streptococcus***	170	24 (14.12)	121 (71.18)	156 (91.76)		14 (8.24)
*Streptococcus acidominimus*	1		1 (100.00)	1 (100.00)		
*Streptococcus agalactiae*	7	1 (14.29)	5 (71.43)	7 (100.00)		
*Streptococcus anginosus*	26	4 (15.38)	25 (96.15)	25 (96.15)		1 (3.85)
*Streptococcus constellatus*	7		7 (100.00)	7 (100.00)		
*Streptococcus cristatus*	1		1 (100.00)	1 (100.00)		
*Streptococcus dysgalactiae*	8		7 (87.50)	7 (87.50)		1 (12.50)
*Streptococcus gordonii*	18	2 (11.11)	17 (94.44)	18 (100.00)		
*Streptococcus mitis/oralis*	38	12 (31.58)	20 (52.63)	28 (73.68)		10 (26.32)
*Streptococcus pneumoniae*	14	2 (14.29)	11 (78.57)	14 (100.00)		
*Streptococcus pyogenes*	1		1 (100.00)	1 (100.00)		
*Streptococcus salivarius*	6	3 (50.00)	4 (66.67)	5 (83.33)		1 (16.67)
*Streptococcus sanguinis*	23		22 (95.65)	22 (95.65)		1 (4.35)
*Streptococcus sinensis*	16			16 (100.00)		
*Streptococcus vestibularis*	4			4 (100.00)		
***Enterococcus***	65	38 (58.46)	60 (92.31)	64 (98.46)	1 (1.54)	
*Enterococcus avium*	1		1 (100.00)	1 (100.00)		
*Enterococcus casseliflavus*	2		1 (50.00)	2 (100.00)		
*Enterococcus faecalis*	19	15 (78.95)	18 (94.74)	19 (100.00)		
*Enterococcus faecium*	41	23 (56.10)	39 (95.12)	40 (97.56)	1 (2.44)	
*Enterococcus gallinarum*	2		1 (50.00)	2 (100.00)		
***Others***	67	28 (41.79)	48 (71.64)	56 (97.01)		2 (2.99)
*Abiotrophia defectiva*	12		4 (33.33)	12 (100.00)		
*Arthrobacter polymorpha*	1			1 (100.00)		
*Bacillus cereus*	2	2 (100.00)	2 (100.00)	2 (100.00)		
*Bacillus subtilis*	1	1 (100.00)	1 (100.00)	1 (100.00)		
*Brevibacterium casei*	2	2 (100.00)	2 (100.00)	2 (100.00)		
*Corynebacterium aurimucosum*	1		1 (100.00)	1 (100.00)		
*Corynebacterium diphtheroides*	2					2 (100.00)
*Corynebacterium glucuronolyticum*	1		1 (100.00)	1 (100.00)		
*Corynebacterium jeikeium*	1			1 (100.00)		
*Corynebacterium mucifaciens*	5	3 (60.00)	4 (80.00)	5 (100.00)		
*Corynebacterium striatum*	6	2 (33.33)	3 (50.00)	6 (100.00)		
*Granulicatella adiacens*	11	6 (54.55)	10 (90.91)	11 (100.00)		
*Lactococcus garvieae*	1	1 (100.00)	1 (100.00)	1 (100.00)		
*Leifsonia aquatica*	1	1 (100.00)	1 (100.00)	1 (100.00)		
*Listeria monocytogenes*	9	6 (66.67)	9 (100.00)	9 (100.00)		
*Micrococcus luteus*	7	3 (42.86)	5 (71.43)	7 (100.00)		
*Mycobacterium avium*	1		1 (100.00)	1 (100.00)		
*Rothia mucilaginosa*	3	1 (33.33)	3 (100.00)	3 (100.00)		
***Gram-negative bacteria***	976	673 (68.95)	918 (94.06)	950 (97.34)	4 (0.41)	22 (2.25)
***Enterobacterales***	721	535 (74.20)	698 (96.81)	712 (98.75)	2 (0.28)	7 (0.97)
*Citrobacter braakii*	2	1 (50.00)	2 (100.00)	2 (100.00)		
*Citrobacter freundii*	8	7 (87.50)	8 (100.00)	8 (100.00)		
*Citrobacter koseri*	5	4 (80.00)	5 (100.00)	5 (100.00)		
*Enterobacter aerogenes*	13	10 (76.92)	12 (92.31)	13 (100.00)		
*Enterobacter agglomerans*	4	1 (25.00)	3 (75.00)	4 (100.00)		
*Enterobacter asburiae*	6	2 (33.33)	5 (83.33)	6 (100.00)		
*Enterobacter cloacae*	28	17 (60.71)	23 (82.14)	23 (82.14)		5 (17.86)
*Enterobacter Kobei*	3	1 (33.33)	2 (66.67)	2 (66.67)	1 (33.33)	
*Escherichia coli*	370	281(75.95)	366 (98.92)	369 (99.73)	1 (0.27)	
*Escherichia fergusonii*	1		1 (100.00)	1 (100.00)		
*Klebsiella oxytoca*	14	9 (64.29)	12 (85.71)	12 (85.71)		2 (14.29)
*Klebsiella planticola*	1	1 (100.00)	1 (100.00)	1 (100.00)		
*Klebsiella pneumoniae*	214	177 (82.71)	209 (97.66)	214 (100.00)		
*Morganella morganii*	4	2 (50.00)	4 (100.00)	4 (100.00)		
*Proteus mirabilis*	9	6 (66.67)	8 (88.89)	9 (100.00)		
*Salmonella SP*	7	3 (42.86)	7 (100.00)	7 (100.00)		
*Serratia marcescens*	32	13 (40.63)	30 (93.75)	32 (100.00)		
***Others***	255	138 (54.12)	220 (86.27)	238 (93.33)	2 (0.78)	15 (5.88)
*Acinetobacter baumannii*	64	42 (65.63)	53 (82.81)	54 (84.38)		10 (15.63)
*Acinetobacter johnsonii*	1		1 (100.00)	1 (100.00)		
*Acinetobacter junii*	3	2 (66.67)	3 (100.00)	3 (100.00)		
*Acinetobacter lwoffii*	1					1 (100.00)
*Aeromonas caviae*	2		2 (100.00)	2 (100.00)		
*Aeromonas hydrophila*	4	2 (50.00)	4 (100.00)	4 (100.00)		
*Brucella*	7	5 (71.43)	7 (100.00)	7 (100.00)		
*Burkholderia cepacia*	7	5 (71.43)	6 (85.71)	6 (85.71)		1 (14.29)
*Burkholderia gladioli*	1	1 (100.00)	1 (100.00)	1 (100.00)		
*Chryseobacterium hominis*	1		1 (100.00)	1 (100.00)		
*Chryseobacterium indologenes*	1	1 (100.00)	1 (100.00)	1 (100.00)		
*Cupriavidus gilardii*	9	8 (88.89)	8 (88.89)	8 (88.89)	1 (11.11)	
*Delftia acidovorans*	1	1 (100.00)	1 (100.00)	1 (100.00)		
*Dialister*	2			2 (100.00)		
*Haemophilus influenzae*	4	2 (50.00)	4 (100.00)	4 (100.00)		
*Haemophilus parainfluenzae*	4	0.00		4 (100.00)		
*Kluyvera ascorbata*	4	2 (50.00)	4 (100.00)	4 (100.00)		
*Moraxella nonliquefaciens*	1		1 (100.00)	1 (100.00)		
*Neisseria elongata ssp elongata*	1			1 (100.00)		
*Neisseria flavescens*	1	1 (100.00)	1 (100.00)	1 (100.00)		
*Ochrobactrum anthropi*	7	1 (14.29)	4 (57.14)	6 (85.71)		1 (14.29)
*Ochrobactrum gallinifaecis*	1		1 (100.00)	1 (100.00)		
*Plesiomonas shigelloides*	4	2 (50.00)	4 (100.00)	4 (100.00)		
*Pseudomonas aeruginosa*	62	49 (79.03)	60 (96.77)	61 (98.39)	1 (1.61)	
*Pseudomonas alcaligenes*	1	1 (100.00)	1 (100.00)	1 (100.00)		
*Ralstonia mannitolilytica*	1		1 (100.00)	1 (100.00)		
*Ralstonia pickettii*	7	1 (14.29)	4 (57.14)	5 (71.43)		2 (28.57)
*Rhizobium radiobacter*	1	1 (100.00)	1 (100.00)	1 (100.00)		
*Sphingomonas paucimobilis*	1	1 (100.00)	1 (100.00)	1 (100.00)		
*Stenotrophomonas maltophilia*	51	10 (19.61)	45 (88.24)	51 (100.00)		
***Fungi***	138	12 (8.70)	84 (60.87)	128 (92.75)	10 (7.25)	
*Candida albicans*	33	1 (3.03)	11 (33.33)	28 (84.85)	5 (15.15)	
*Candida glabrata*	20	2 (10.00)	6 (30.00)	17 (85.00)	3 (15.00)	
*Candida parapsilosis*	39		29 (74.36)	38 (97.44)	1 (2.56)	
*Candida tropicalis*	42	8 (19.05)	34 (80.95)	41 (97.62)	1 (2.38)	
*Pichia norvegensis*	1		1 (100.00)	1 (100.00)		
*Saccharomyces cerevisiae*	1	1 (100.00)	1 (100.00)	1 (100.00)		
*Trichosporon asahii*	2		2 (100.00)	2 (100.00)		
***Anaerobic bacteria***	30	15 (50.00)	28 (93.33)	29 (96.67)	1 (3.33)	
*Anaerobiospirillum succiniciproducens*	2		2 (100.00)	2 (100.00)		
*Bacteroides fragilis*	13	4 (30.77)	13 (100.00)	13 (100.00)		
*Clostridium clostridiiform*	3	3 (100.00)	3 (100.00)	3 (100.00)		
*Clostridium innocuum*	4	2 (50.00)	3 (75.00)	3 (75.00)	1 (25.00)	
*Prevotella bivia*	5	5 (100.00)	5 (100.00)	5 (100.00)		
*Prevotella buccae*	1	1 (100.00)	1 (100.00)	1 (100.00)		
*Propionibacterium acnes*	2		1 (50.00)	2 (100.00)		
**Total**	2,032	1,136 (55.91)	1,780 (87.60)	1,956 (96.26)	23 (1.13)	53 (2.61)

**Table 2:** The percentage of concordant results between direct identification and conventional method among 2,032 monomicrobial blood cultures.

Group of Microorganism	Conventional method (no. of isolates)	Direct method		
		No. (%) log(score) of:		
		Score ≥2.000	Score ≥1.700	Score ≥1.400
Gram-positive bacteria	888	49.10%	84.46%	95.61%
Gram-negative bacteria	976	68.95%	94.06%	97.34%
Fungi	138	8.70%	60.87%	92.75%
Anaerobic bacteria	30	50%	93.33%	96.67%
Total	2,032	55.91%	87.60%	96.26%

With the optimized protocol, 94.06% (918/976) gram-negative isolates were identified with a score ≥1.700. A lower percentage was reached to the species level, which is 68.95% (673/976). There were 721 *Enterobacterales* strains in total, and 96.81% (698/721) were identified with scores higher than 1.700. *Nonfermenting gram-negative bacilli*, mainly consists of *Acinetobacter*, *Pseudomonas*, and *Stenotrophomonas*, also had a high compliance rate with the results obtained from the conventional phenotypic identification, which is 89.07% (163/183) (**Figure 3C**).

For 888 gram-positive organisms, the scores of 84.46% (750/888) isolates ≥1.700. There are 88.91% (521/586) *Staphylococcus* were identified to the genus level. A score ≥1.700 was obtained for 97.61% (204/209) of *Staphylococcus aureus*. The 121 isolates of *Streptococcus* exhibited 71.18% (121/170) concordance with the results of conventional laboratory culture-dependent identification. *Streptococcus anginosus, Streptococcus sanguinis*, and *Streptococcus gordonii* showed high confidence identification rates, which were 96.15% (25/26), 95.65% (22/23), and 94.44% (17/18) respectively. Also, *Enterococcus* presented excellent identification with 92.31% (60/65) to the genus level. *Enterococcus faecium* and *Enterococcus faecalis* were correctly identified with 95.12% (39/41) and 94.74% (18/19) with scores higher than 1.700 (**Figure 3D**).

Among the fungi, 84 out of 138 samples (60.87%) were able to be identified at the genus level with the optimized protocol. There were seven different species of fungi that had been identified in our study. After extra lysis, *Candida tropicalis* had a concordance rate of 80.95% (34/42) with scores higher than 1.700, and 74.36% (29/39) *Candida parapsilosis* was identified to genes level. Low concordance rate was associated with *Candida albicans* (33.33%, 11/33) and *Candida glabrata* (30%, 6/20).

Seven species of anaerobic bacteria were included in our study. 93.33% (28/30) anaerobic bacteria were secured to the genus level. All of our 21 strains of gram-negative anaerobes were successfully identified to the genus level. Only two strains of gram-positive anaerobes failed to obtain reliable results. One (of two) *Propionibacterium acnes* got a score of 1.659, nearly to 1.700. One (of four) *Clostridium innocuum* got a score under 1.400.

The pretreatment time including lytic, washing, and re-suspension bacteria is about 10 min, and the identification time for MALDI-TOF MS analysis is about 2 min. Hence, only 12 min is needed for this protocol. For the identification of fungi, due to the requirement for additional extraction steps, the sample processing time is about 10 min longer than that of bacteria.

## Discussion

We described a rapid and simplified protocol for direct identification of microorganisms from positive blood cultures with MALDI-TOF MS in this study. The efficacy of the protocol was validated with 2032 isolates. *Enterobacterales* (96.81%), *Enterococcus* (92.31%), *Nonfermenting bacilli* (89.07%), *Staphylococcus* (88.91%), and other general organisms causing BSIs were excellently identified. It is worth noting that, compared to existing methods, including similar lysis and centrifugation approach, our protocol shows superiority. The identification success rates of our method are similar to more complicated methods but higher than most of the nonmodified methods (**Table S1**) (Campigotto et al., 2018; Lin et al., 2018; Pan et al., 2018; Tsuchida et al., 2018; Azrad et al., 2019). We noticed that Simon’s research group has used the lysis and centrifugation method for blood culture broth extraction. Among 632 blood cultures, they reached a concordance rate of 80% with the conventional method when the log (score) threshold was ≥1.500 (Simon et al., 2019). However, they didn’t include fungi in their work. Unlike Simon’s work, we have developed an effective extra extraction process for fungi, which is the contribution of our work. Besides, our protocol remarkably shortened the duration of the processing, and less amount of broth is needed (**Table S2**) (Loonen et al., 2012; Robinson and Ussher, 2016; Yonetani et al., 2016; Caspar et al., 2017). It is also easier to be integrated into clinical laboratories because it is economic and requires fewer personnel.

The species identification success rate of gram-negative aerobes was generally high. However, we noticed that five (of 28) strains of *Enterobacter cloacae* were incorrectly identified as *Enterobacter hormaechei*, which is in line with the previous study (Juiz et al., 2012). Meanwhile, the 10 discordant results of *Acinetobacter baumannii* consisted of six *Acinetobacter nosocomialis* and four *Acinetobacter pittii*. Several studies have reported that MALDI-TOF MS had defects in the species-level identification of *Acinetobacter spp* as well as *Enterobacter spp*, because their species are similar in phenotype and protein profile (Turton et al., 2010; Lee et al., 2011; Juiz et al., 2012; Jeong et al., 2016).

The consistency rate of our protocol with the conventional method for gram-positive bacteria (84.46%) is still lower than for gram-negative bacteria (94.06%). The thicker peptidoglycan cell walls of gram-positive bacteria can render these bacteria more resistant to cleavage than their gram-negative counterparts, resulting in poor MALDI-TOF MS profiles (Risch et al., 2010; Clark et al., 2013). Nine of ten mis-identified *Streptococcus mitis/oralis* were erroneously identified as *Streptococcus pneumoniae* in our study. Like other protocols, it is difficult to distinguish *Streptococcus pneumoniae* with *Streptococcus mitis/oralis*, which are closely related species of viridans group *Streptococcus* (VGS) (Yonetani et al., 2016; Simon et al., 2019). VGS have the similar 16sRNA and a close compose of protein (Yonetani et al., 2016). Although the ability of MALDI-TOF MS for identifying VGS into species-level remains controversial, the accuracy for identification to the group level was generally acceptable (Loonen et al., 2012; Su et al., 2018). Additional biochemical tests, such as bile solubility test and optochin susceptibility test could be utilized to help to discriminate *Streptococcus pneumoniae* from *Streptococcus mitis/oralis*.

What's more, we have been able to successfully identify 60% of fungi from blood cultures with extra extraction, which is higher than some studies (Vecchione et al., 2018; Azrad et al., 2019; Wu et al., 2019). It has been reported that the cut-off value could be lowered down to 1.400 without compromising accuracy (Lagace-Wiens et al., 2012; Saffert et al., 2012; Simon et al., 2019). When the cut-off value was not taken into account, all results of fungal samples obtained from our protocol were successfully matched with the results from the conventional method. As reported, the thick cell wall of fungi is one of the predominant reasons that hinder the identification (Prod’hom et al., 2010). The results obtained from this study indicated that extra lysis helps disturb the cell wall and liberate intracellular proteins. We noticed that some results of fungal samples with a score ≤1.700 obtained ten correct results after analyzed by Microflex LT, but with low bacterial load. Insufficient biomass of proteins would make it hard to obtain peaks of sufficient intensity for MS analysis (De Bruyne et al., 2011).

Due to its stringent cultivation requirements, anaerobes are difficult to be cultured and identified in clinical laboratories by conventional approaches. It should be noticed that the correct identification of anaerobes by our method was 93.33% (28/30), indicating that this method is reliable for directly identifying anaerobes.

The seven strains of *Brucella* could not be successfully tested at first because the Species database that the manufacturer provided did not contain the *Brucella* strains. After introducing the spectra of respective *Brucella* into the database, all tested *Brucella* were explicitly identified at the genus level using our protocol. Therefore, optimizing the libraries of information and perfecting the database is useful to improve the identification accuracy of MALDI-TOF MS (Veloo et al., 2018; Li et al., 2019).

This study provides supporting evidence for the direct identification of microorganisms from positive blood cultures by using MALDI-TOF MS, but there are several limitations. A larger-scale test is needed to obtain more accurate information. Although more than 2,000 isolates were included in this study, the sample sizes of fastidious bacteria, fungi, and anaerobes were relatively small. Besides, this protocol has been only tested in one clinical microbiology laboratory, the repeatability and reproducibility of the protocol need to be assessed. The strict and systematic training for operators should be conducted to eliminate the operative difference and reduce analytical errors (Li et al., 2019; Luethy and Johnson, 2019).

Our further study may focus on creating more user-defined databases, directly antimicrobial susceptibility testing, and detecting virulence factors. All of those studies will facilitate the continuing research on the clinical application of MALDI-TOF MS.

In conclusion, this easy-to-use and cost-effective protocol can accurately identify 87.60% of microorganisms from blood cultures in 20 min. It could remarkably shorten the TAT of BSIs diagnosis and reduce morbidity and mortality of patients.

## Data Availability Statement

The original contributions presented in the study are included in the article/**Supplementary Material**. Further inquiries can be directed to the corresponding author.

## Author Contributions

CJ and YD conceptualized and designed the research. CJ, YD, XX, and DL performed the experiments. CJ, YD, XY, WC, LT, and MH carried out the data analysis. CJ and YD wrote the manuscript. All authors contributed to the article and approved the submitted version.

## Funding

This work was supported by the National Nature Science Foundation of China (81701577) and the Natural Science Foundation of Hunan Province of China (2018JJ2559).

## Conflict of Interest

The authors declare that the research was conducted in the absence of any commercial or financial relationships that could be construed as a potential conflict of interest.
