# Supplementary material for: Evaluation of a Rapid and Simplified Protocol for Direct Identification of Microorganisms From Positive Blood Cultures by Using Matrix Assisted Laser Desorption Ionization Time-of-Flight Mass Spectrometry (MALDI-TOF MS)
Source: Front Cell Infect Microbiol. 2021 Mar 11;11:632679. doi: 10.3389/fcimb.2021.632679 (PMC7990877; doi:10.3389/fcimb.2021.632679)
Supplement: Supplementary file 1 [file Table_1.docx]

| **Author** | **Year** | **Bacteria** | **Number of isolates** | **Sepsityper** | **Centrifugation** | **In-house Saponin** | **Lysis-extraction** |
| --- | --- | --- | --- | --- | --- | --- | --- |
| Pan, H. W., et al. | 2018 | G^+^  G^-^ | 72  57 |  |  |  | 88.89%  92.98% |
| Tsuchida, S., et al. | 2018 | G^+^  G^-^ | 67  50 | 76.12%  98% |  |  |  |
| Lin, J. F., et al. | 2018 | G^+^  G^-^ | 138  180 |  | 92.03%  93.89% |  |  |
| Campigotto, A., et al. | 2018 | G^+^  G^-^ | 64  57 |  |  |  | 90.63%  93.60% |
| Azrad, M., et al. | 2019 | G^+^  G^-^ | 88  79 | 57%  92% |  | 38%  52% |  |
| This study | 2020 | G^+^  G^-^ | 888  976 |  |  |  | 84.46%  94.06% |

**Table S1.** Published reports on the percentage of correct identification by MALDI-TOF MS using Sepsityper, centrifugation, in-house saponin and our method (log (score) of ≥1.700).

| **Table S2.** Comparison of four different methods in the cost, time and amount of broth. | | | |
| --- | --- | --- | --- |
| **Test** | **Time (minute per test)** | **Cost ($NZD per test)** | **Amount of broth**  **(ml per test)** |
| MALDI Sepsityper kit ™ (Yonetani et al., 2016) | 30 | $7 | 1 |
| In-house Saponin method (Yonetani et al., 2016) | 30 | $0.5 | 1 |
| Centrifugation method  (Robinson and Ussher, 2016) | 30-45 | ＜$0.10 | 6 |
| Our method | 10-20 | ＜$0.10 | 0.2 |
